# Non-transfusion dependent hemoglobin E/β thalassemia had high prevalence of vitamin D deficiency than more severe patients who received regular blood transfusion and iron chelation therapy

**DOI:** 10.1186/1687-9856-2013-S1-P165

**Published:** 2013-10-03

**Authors:** Pairunyar Nakavachara, Katharee Chaichanwattanakul, Vip Viprakasit

**Affiliations:** 1 Division of Pediatric Endocrinology, Siriraj Hospital, Mahidol University, Bangkok, Thailand; 2 Division of Pediatric Hematology and Oncology, Department of Pediatrics, Siriraj Hospital, Mahidol University, Bangkok, Thailand

## 

Hb E/β thalassemia is the most common β thalassemia syndrome in Asia-Pacific due to a high prevalent of Hb E and β thalassemia genes. Management of this condition can be cumbersome due to its clinical heterogeneity and various disease severity ranging from severe end in which patients are transfusion dependent thalassemia (TD) similar to that of β thalassemia major (TM) to moderate and mild severity which are non-transfusion dependent thalassemia (NTDT) akin to β thalassemia intermedia. Although, clinical diagnosis of Hb E/β thalassemia might be identical, the natural history and disease related complications can vary greatly due to different baseline severity and management received. In this study, we evaluated vitamin D deficiency, one of the most common endocrine complications described in TM, in Thai HbE/β thalassemia children who were TD and NTDT.

109 children aged 5.9 to 14.1 years with HbE/β-thalassemia were enrolled. 60 patients who received no or occasional transfusion were classified into NTDT group. While 49 patients who received regular transfusion every 3 weeks to keep pre-transfusion Hb at 9-10 g/dL were classified into TD group. All TD patients except two received iron chelation. Blood samples were collected to determine hemoglobin, serum ferritin and 25-OHD levels.

Mean Hb of NTDT was lower than TD patients (8.1+1.0 vs. 10.0+1.0 g/dL, P<0.001). In contrast, TD had higher mean serum ferritin than NTDT patients (4104+2336 vs. 347+488 ng/mL, P<0.001). However, mean serum 25-OHD of NTDT was lower than in TDT patients (22.7+5.2 vs. 25.0+6.1 ng/mL, P=0.043). Moreover, the percentage of patients who were vitamin D deficient (serum 25-OHD < 20 ng/mL) in NTDT was higher than TD patients (33.3% vs. 12.2%, P=0.01). We found no correlation between serum 25-OHD vs. ferritin and Hb levels.

Although patients with HbE/b thalassemia do not require regular blood transfusion due to their ‘milder’ clinical severity, they remain at high risk of having vitamin D deficiency. A better standard of care for these neglected patients is in an urgent need and a regular monitoring of serum 25-OHD with adequate vitamin D supplementation for deficient patients is highly recommended for all Hb E/b thalassemia regardless of their clinical severity.

